# The Dynamic Role of Microglia and the Endocannabinoid System in Neuroinflammation

**DOI:** 10.3389/fphar.2021.806417

**Published:** 2022-02-04

**Authors:** Alexander P. Young, Eileen M. Denovan-Wright

**Affiliations:** Department of Pharmacology, Dalhousie University, Halifax, NS, Canada

**Keywords:** neuroinflammation, endocannabinoid system, CB_2_ receptor, microglia, MAPK signaling, heteromer, GPCR (G protein coupled receptor)

## Abstract

Microglia, the resident immune cells of the brain, can take on a range of pro- or anti-inflammatory phenotypes to maintain homeostasis. However, the sustained activation of pro-inflammatory microglia can lead to a state of chronic neuroinflammation characterized by high concentrations of neurotoxic soluble factors throughout the brain. In healthy brains, the inflammatory processes cease and microglia transition to an anti-inflammatory phenotype, but failure to halt the pro-inflammatory processes is a characteristic of many neurological disorders. The endocannabinoid system has been identified as a promising therapeutic target for chronic neuroinflammation as there is evidence that synthetic and endogenously produced cannabinoids temper the pro-inflammatory response of microglia and may encourage a switch to an anti-inflammatory phenotype. Activation of cannabinoid type 2 (CB_2_) receptors has been proposed as the mechanism of action responsible for these effects. The abundance of components of the endocannabinoid system in microglia also change dynamically in response to several brain pathologies. This can impact the ability of microglia to synthesize and degrade endocannabinoids or react to endogenous and exogenous cannabinoids. Cannabinoid receptors also participate in the formation of receptor heteromers which influences their function specifically in cells that express both receptors, such as microglia. This creates opportunities for drug-drug interactions between CB_2_ receptor-targeted therapies and other classes of drugs. In this article, we review the roles of pro- and anti-inflammatory microglia in the development and resolution of neuroinflammation. We also discuss the fluctuations observed in the components of the endocannabinoid in microglia and examine the potential of CB_2_ receptors as a therapeutic target in this context.

## Introduction

Neuroinflammation is characterized by sustained activation of microglia which release toxic cytokines that cause widespread damage to the brain. Microglia are recognized as the resident immune cells of the brain and have been identified as active propagators of neuroinflammation throughout the progression of several neurodegenerative diseases (Perry et al., 2010). At rest, microglia secrete neurotrophins and clear debris to support the maintenance of normal brain function (Cherry et al., 2014a). These unreactive microglia survey their environment using scavenger receptors to sense disruptions to local homeostasis (Nimmerjahn et al., 2005). Microglia detect soluble factors released by neurons, astrocytes, other microglia, and infiltrating peripheral immune cells and may transition toward either an activated M1 (pro-inflammatory) or M2 (anti-inflammatory) phenotype to maintain homeostasis (Chhor et al., 2013). M1 microglia mediate host defense and are characterized by regulated phagocytic activity and the release of pro-inflammatory cytokines such as interleukin (IL)-1β and tumour necrosis factor alpha (TNFα) (Chhor et al., 2013). If these cytokines reach sufficient concentrations, they will trigger neuronal signaling cascades that cause cell impairment or necrosis (Neumann et al., 2002; Bachiller et al., 2018). In a healthy brain, the inflammatory process will halt before this occurs and M1 microglia will transition toward a more anti-inflammatory M2 phenotype to release anti-inflammatory cytokines, clear debris from dead cells, promote angiogenesis, and deposit extracellular matrix (Varin and Gordon, 2009; Cherry et al., 2014b). However, failure to halt the inflammatory process and engage M2 microglia is a common characteristic of several neurological disorders (Cherry et al., 2014b).

Microglia possess the necessary components required to synthesize, degrade, and respond to extracellular endocannabinoids (Stella, 2009; Stella, 2010). The endocannabinoid system comprises the cannabinoid type 1 (CB_1_) and type 2 (CB_2_) receptors, the endogenous ligands anandamide (AEA) and 2-arachidonylglycerol (2-AG), as well as the enzymes that regulate their production (Lu and Mackie, 2016). Anandamide was the first identified endocannabinoid which is known to bind CB_1_ receptors as well as CB_2_ receptors with relatively low affinity (Devane et al., 1992; Felder et al., 1993). AEA is synthesized by the enzyme N-acyl phosphatidylethanolamine-specific phospholipase D (NAPE-PLD) and degraded by fatty acid amide hydrolase (FAAH). The second identified endocannabinoid was 2-arachidonoylglycerol (2-AG) which also activates both CB_1_ and CB_2_ receptors (Mechoulam et al., 1995; Sugiura et al., 1995; Stella et al., 1997). In human serum, 2-AG is up to 100-fold more abundant than AEA (Hillard et al., 2012). 2-AG is synthesized by diacylglycerol lipase (DAGL) and degraded primarily by monoacylglycerol lipase (MAGL) as well as alpha/beta-hydrolase domain (ABHD) containing enzymes such as ABHD6 and ABHD12 (Di Marzo et al., 1994; Cravatt et al., 2001). Although both endocannabinoids have effects on analgesia, AEA has greater effects on depression and anxiety whereas 2-AG appears to contribute more to the effects on movement and temperature regulation (Kathuria et al., 2003; Gobbi et al., 2005; Long et al., 2009). When both endocannabinoids are elevated through dual blockade of FAAH and MAGL, the effects mimic that of Δ9-tetrahydrocannabinol (Δ^9^-THC) from *Cannabis* (Long et al., 2009; Alger and Kim, 2011).

Cannabinoid receptors are G protein-coupled receptors (GPCRs) that typically couple to Gα_i_ but have been observed to couple to Gα_o_ and Gα_s_ under some circumstances (Glass and Felder, 1997; Howlett et al., 2002; Saroz et al., 2019). CB_1_ receptors are abundant in central neurons and inhibit transmitter release upon activation (Howlett et al., 2002). CB_2_ receptors display a distinct pharmacological profile and are more abundant in peripheral immune cells as well as in microglia (Galiègue et al., 1995; Howlett et al., 2002; Stella, 2009). CB_1_ receptors generally exert the psychoactive effects of Δ^9^-THC, whereas CB_2_ receptors primarily mediate the immunosuppressive and anti-inflammatory effects of select cannabinoid molecules (Bouaboula et al., 1993; Lynn and Herkenham, 1994; Galiègue et al., 1995; Marsicano and Lutz, 1999). Pro-inflammatory and anti-inflammatory microglial phenotypes exhibit changes in the concentration of endocannabinoids as well as differences in the enzymatic machinery to synthesize and metabolize them (Maresz et al., 2005; Mecha et al., 2015). Furthermore, the quantities of the cannabinoid receptors have been observed to fluctuate widely in response to different pro- and anti-inflammatory stimuli. Current data that describe which components of the endocannabinoid system are upregulated or downregulated in each phenotype is useful to understand that the endocannabinoid system is a moving target in the context of neuroinflammation.

Neuroinflammation is a hallmark of aging as well as neurodegenerative diseases including Alzheimer’s disease (AD), Parkinson’s disease (PD), and Huntington’s disease (HD) (Guzman-Martinez et al., 2019). Each of these neurodegenerative diseases are characterized by overactivation of microglia and have a neuroinflammatory component which could be a common target for therapeutics. The endocannabinoid system has been identified as a promising source of targets for the treatment of such chronic neuroinflammation (Pacher et al., 2006; Ashton and Glass, 2007; Saito et al., 2012). However, the molecular mechanisms that underlie the success of these treatments have not been clearly defined (Tanaka et al., 2020). Cannabinoids appear to dampen the pro-inflammatory microglial phenotype via multiple signaling pathways to regulate the transition from a resting to an anti-inflammatory microglial phenotype. To add an additional layer of complexity, cannabinoid receptors have recently been found to form oligomeric receptor complexes which respond differently to cannabinoids relative to the individual receptors; this may allow for unanticipated drug-drug interactions among CB_2_ receptor agonists and other cannabinoids or other classes of drugs that target microglia. In this review, we discuss the roles of pro- and anti-inflammatory microglia in the development and resolution of neuroinflammation. We also discuss the fluctuations observed in the components of the endocannabinoid in microglia and examine the potential of CB_2_ receptors as a therapeutic target in this context.

## Microglial Phenotypes and the Endocannabinoid System

**FIGURE 1:**
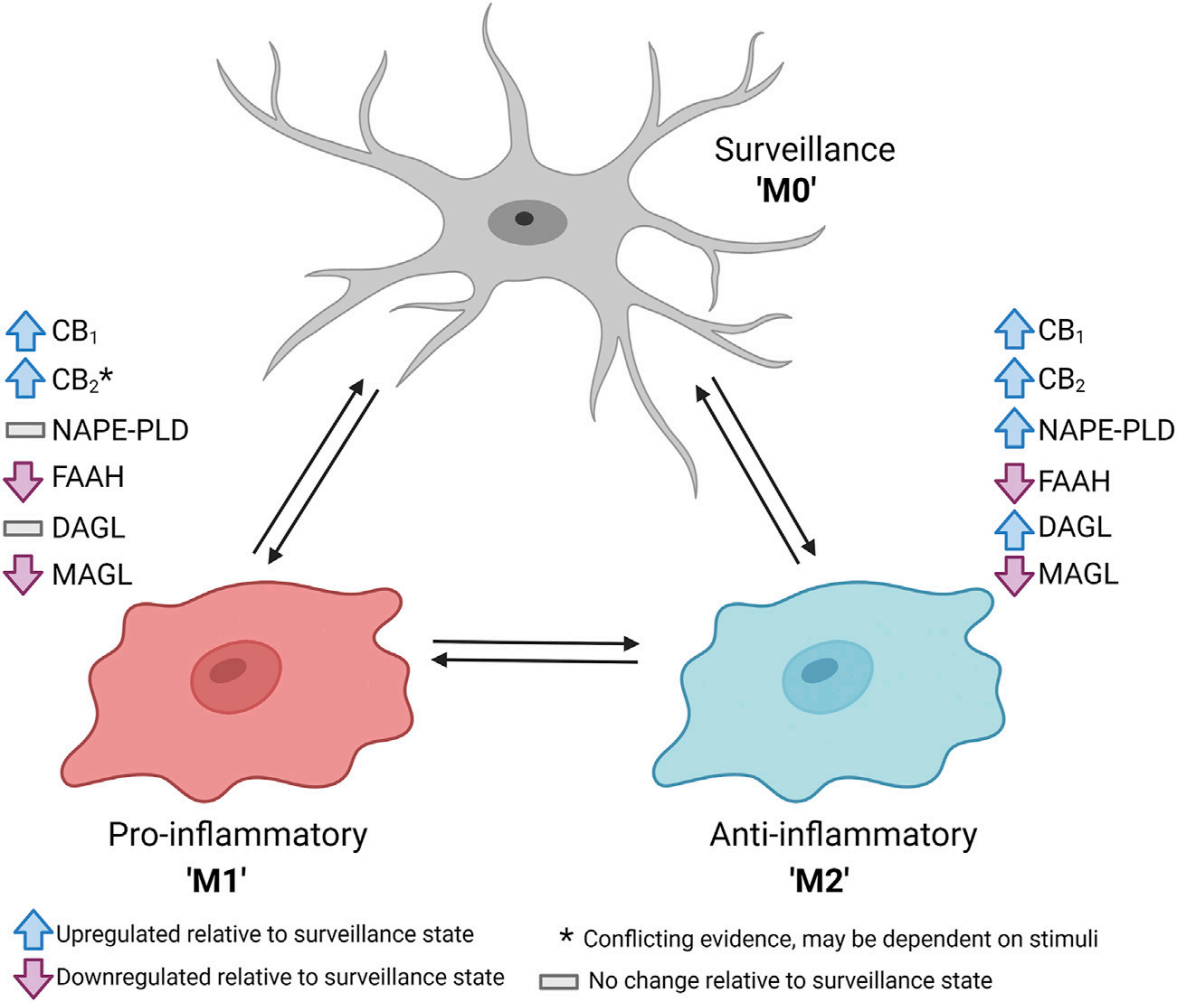
Schematic summary of changes in the components of the endocannabinoid system upon dynamic shift from unreactive or surveillance (M0) phenotype to a pro-inflammatory (M1) or anti-inflammatory (M2) phenotype. Data derived primarily from Maresz et al. (2005), Mecha et al. (2015), and Navarro et al. (2018). Figure created with BioRender.

The microglial endocannabinoid system changes substantially among different phenotypes (Figure 1). At rest, microglia engage in several tasks including surveillance of the brain parenchyma and the maintenance of synapse function, and the abundance of CB_1_ and CB_2_ receptors is expected to be relatively low (Nimmerjahn et al., 2005; Stella, 2010). Early reports indicated that CB_1_ and CB_2_ receptor mRNA was undetectable within healthy brain tissue lysate or in isolated resting microglia (Munro et al., 1993; Galiègue et al., 1995; Schatz et al., 1997; Griffin et al., 1999; McCoy et al., 1999; Sugiura et al., 2000; Carlisle et al., 2002). However, other reports have indicated that resting microglia expressed both cannabinoid receptors, although perhaps only in trace amounts (Núñez et al., 2004; Navarro et al., 2018). Unreactive glia have been observed to release both AEA and 2-AG at a ratio of roughly 1:100 (Mecha et al., 2015; Araujo et al., 2019). If endocannabinoids released by resting microglia interacted with local synapses, CB_1_ receptors could be activated to inhibit transmitter release from the pre-synaptic neurons *via* modulation of intracellular calcium, cyclic AMP, and inwardly rectifying potassium currents (Howlett et al., 2002). However, it is still uncertain whether endocannabinoids released specifically by microglia directly influence the activity of local synapses.

Under conditions of neuroinflammation, microglia engage in a pro-inflammatory M1 phenotype which includes several changes to their endocannabinoid function. Maresz et al. (2005) initially observed that CB_2_ receptor mRNA was upregulated by 100-fold in the central nervous system (CNS) of mice with experimental autoimmune encephalitis. The pattern was consistent in primary mouse microglia treated with interferon-γ (IFNγ). These findings were replicated in immortalized N9 microglia as stimulation with IFNγ and lipopolysaccharide (LPS) caused a 12-fold increase in CB_2_ receptor mRNA (Navarro et al., 2018). Conversely, primary rat microglia stimulated with LPS for 6 h exhibited a global downregulation of the components of the endocannabinoid system, including mRNA for CB_1_ and CB_2_ receptors, NAPE-PLD and FAAH, as well as DAGLα, DAGLβ, and MAGL (Mecha et al., 2015). After 24 h, FAAH and MAGL remained depressed, but the other components returned to baseline which may be indicative of a compensatory mechanism to favour synthesis of endocannabinoids following pro-inflammatory insult. Taken together, it appears that the regulation of CB_2_ receptors and other components of the endocannabinoid system in microglia under pro-inflammatory conditions may depend on the type of stimuli or the length of time exposed to such conditions. Interestingly, recent RNA sequencing data revealed that microglia isolated from CB_2_ receptor knockout mice failed to transition to an M1 phenotype in response to IFNγ and LPS (Reusch et al., 2021). This is indicative of potential crosstalk between CB_2_ receptor-mediated signaling and the effects of toll-like or IFN receptors. Thus, constitutive CB_2_ receptor activation may facilitate the initial transition to a pro-inflammatory phenotype.

To assist with the resolution of neuroinflammation, microglia take on an anti-inflammatory M2 phenotype which includes unique changes to microglial endocannabinoid function (Tanaka et al., 2020). Mecha et al. (2015) determined that rat microglia treated with IL-4 and IL-13 for 6 h had an enhanced abundance of mRNA for CB_2_ receptors and DAGLα. After 24 h, mRNA for CB_1_ receptors and NAPE-PLD became elevated, and DAGLα had returned to baseline. These cells also exhibited reduced mRNA for FAAH and MAGL in both the 6- and 24-h treatment groups. Furthermore, these M2 microglia also released more AEA and 2-AG (Mecha et al., 2015). This indicates that microglia in an M2 phenotype promote the synthesis of both endocannabinoids and have a lower quantity of degradative enzymes relative to the resting or M1 phenotypes. The same authors also determined that treatment of microglia with endocannabinoids *in vitro* caused an upregulation of both CB_1_ and CB_2_ receptor mRNA abundance (Mecha et al., 2015). Activation of either CB_1_ or CB_2_ receptors by endocannabinoids has also been demonstrated to induce a shift toward an M2 phenotype in microglia, with upregulation of mRNA and protein for anti-inflammatory markers such as Arg-1 and SOCS-3 (Correa et al., 2010; Correa et al., 2011; Mecha et al., 2015). Taken together, the current evidence suggests that endocannabinoids promote microglia to shift toward an M2 phenotype which contributes to a feed-forward loop to upregulate cannabinoid receptor expression and release more endocannabinoids. Finally, microglia treated with IL-4 and IL-13 exhibited a substantial increase in Arg-1 mRNA and protein, but the effect was fully blocked by selective antagonists for either CB_1_ receptors (AM251) or CB_2_ receptors (AM630) (Mecha et al., 2015). Thus, constitutive activity of both CB_1_ and CB_2_ receptors may be required to enable the transition from an unreactive to an M2 phenotype.

## Microglial Phenotypes and Neuroinflammation With Disease and Aging

Changes in microglial phenotype have been observed in neurodegenerative diseases such as AD, PD, and HD as well as with normal aging. Each of these states also exhibit unique changes to the endocannabinoid system which includes fluctuations in global AEA and 2-AG concentrations as well as changes in CB_1_ and CB_2_ receptor abundance (Supplementary Table S1). Although the components of the endocannabinoid system vary in microglia among pathologies, the cannabinoid receptors have shown promise as therapeutic targets for the treatment of neuroinflammation and neurodegeneration.

### Alzheimer’s Disease

AD is characterized by the aggregation of amyloid-beta (Aβ) that form extracellular plaques and accumulation of intracellular tau protein that form neurofibrillary tangles (Bloom, 2014). The buildup of cellular plaques and tangles results in the death of affected neurons with a subsequent decline in cognitive function (Selkoe and Hardy, 2016). The cause of AD was initially ascribed to insufficient clearance of Aβ aggregates and hyperphosphorylated tau protein (Murphy and LeVine, 2010). In AD mice, microglia are observed near Aβ plaques at five-fold the normal density, the purpose of which has been proposed to be clearance of Aβ by phagocytosis (Frautschy et al., 1998; D’Andrea et al., 2004). However, current evidence indicates that microglia do not influence the size or number of Aβ plaques in the late stages of AD (Spangenberg et al., 2016). More recent data has demonstrated that ablation of microglia in AD mice using a colony-stimulating factor 1 (CSF1) antagonist prevented the formation of these plaques which indicates that microglia may not be responsible for Aβ clearance but may in fact contribute to the initial deposition of Aβ plaques (Spangenberg et al., 2019). Furthermore, the accumulation of activated non-plaque associated microglia may lead to a sustained localized release of proinflammatory cytokines including IL-1β, IL-6, NO, and TNFα which are also neurotoxic (Benzing et al., 1999; Abbas et al., 2002; Wang et al., 2015). The increased concentration of these cytokines could exacerbate the accumulation of Aβ and produce further damage to the brain (Hickman et al., 2008). The severity of AD dementia has shown to be positively correlated with markers of pro-inflammatory microglial activation (Nordengen et al., 2019). Although originally thought of as a secondary effect of plaque formation, neuroinflammation is now understood to contribute equally to AD progression compared to the canonical protein aggregates (Zhang et al., 2013; Heneka et al., 2015).

Mouse models of AD have consistently demonstrated specific changes in the endocannabinoid system, including upregulation of CB_2_ receptors and dysregulation of 2-AG metabolism (Mulder et al., 2011; Cristino et al., 2020). Enhanced CB_2_ receptor-like immunoreactivity was found localized within plaque-associated microglia in human AD tissue (Benito et al., 2003). The same pattern of elevated CB_2_ receptor-like immunoreactivity in human AD brains was later reported (Halleskog et al., 2011). Western blots from human AD brain lysate later corroborated that CB_2_ receptor protein was elevated in the frontal cortex (Solas et al., 2013). Rats that received intracerebral injection of 30 ng of Aβ exhibited 2.7-fold increased CB_2_ receptor mRNA abundance (Esposito et al., 2007). Aso et al. (2013) identified that the APP/PS1 mouse model of AD exhibited 1.4-fold increased CB_2_ receptor mRNA abundance. Examinations of human AD brains have shown no change in total protein for NAPE-PLD or FAAH (Mulder et al., 2011), although FAAH activity may be selectively upregulated in the plaque-associated glia (Benito et al., 2003). Conversely, substantial changes have been observed in the metabolic enzymes for 2-AG (Mulder et al., 2011). Tissue from human AD brains revealed a positive correlation between disease stage and upregulation of DAGL and MAGL, with no alteration in ABHD6 (Mulder et al., 2011). Isolated membrane and cytosolic fractions from this tissue also exhibited a faster rate of 2-AG degradation compared to control tissue. Concentrations of AEA have been positively correlated with cognitive function in AD patients but negatively correlated with abundance of Aβ_42_ which indicates that AD is also associated with dysregulated AEA production (Jung et al., 2012). Ultimately, brains afflicted with AD exhibit reduced endocannabinoid signaling which is likely caused by enhanced degradation of endocannabinoids without a compensatory increase in the synthetic enzymes.

CB_2_ receptor activation has demonstrated potential benefits in several models of AD to dampen neuroinflammation and improve cognition (Martín-Moreno et al., 2012; Cassano et al., 2017; Li et al., 2019). The nonselective cannabinoid agonist, WIN55212-2, dampened the inflammatory response in rats that received hippocampal injection of Aβ (Fakhfouri et al., 2012). APP/PS1 mice administered a selective CB_2_ receptor agonist, JWH-133, exhibited a partial rescue of cognitive deficits as determined by an active avoidance test and a V-maze memory test (Aso et al., 2013). This improvement in cognitive performance was accompanied by a reduction in the pro-inflammatory markers IL-1β, IL-6, and TNFα. However, the drug was only effective when administered at the pre-symptomatic stage. Furthermore, JWH-133 had no effect on the quantity of Aβ in the brain (Aso et al., 2013). Mice that received JWH-133 also exhibited greater numbers of microglia that expressed elevated levels of IL-6 and IL-10 which was indicative of immunoregulatory activity (Aso et al., 2013; Chhor et al., 2013). Activation of microglial CB_2_ receptors has also been shown to stimulate phagocytosis of Aβ *in vivo* and *in vitro* (Tolón et al., 2009; Aso et al., 2016). Thus, activation of microglial CB_2_ receptors appears to serve a dual purpose to enhance phagocytosis of Aβ plaques and dampen neuroinflammation.

### Parkinson’s Disease

PD is characterized by motor dysfunction due to damage to dopaminergic neurons of the nigrostriatal pathway. Activated M1 microglia have been determined to be closely associated with neuron damage in human PD brains (McGeer et al., 1988). Imamura et al. (2003) found that the abundance of CD54/CD11a^+^ microglia was correlated with progressive neurodegeneration in the substantia nigra. Increased proportions of activated microglia were also located in the caudate nucleus, hippocampus, transentorhinal cortex, cingulate cortex, and temporal cortex compared to the healthy control subjects. These activated microglia were also positive for TNFα and IL-6 which was indicative of an M1 phenotype. Subsequent studies have supported the elevated presence of M1 microglia in PD brains (Ouchi et al., 2005; Gerhard et al., 2006). However, quantities of activated microglia were not necessarily correlated with clinical severity (Gerhard et al., 2006). Dopaminergic neurons of the midbrain have been shown to be especially sensitive to the toxic effects of microglial cytokines including TNFα (McGuire et al., 2001). Thus, this population of neurons is highly susceptible to severe damage triggered by neuroinflammation that is characterized by the perpetuating cycle of neuron death with subsequent reactive microglial activation and cytokine release.

There are conflicting reports with respect to fluctuating levels of CB_1_ and CB_2_ receptors in PD. Elevated CB_1_ receptor mRNA has been observed in the caudate putamen but not the substantia nigra of human PD brains (Navarrete et al., 2018). In non-human primates subjected to 1-methyl-4-phenyl-1,2,3,6-tetrahydropyridine (MPTP)-induced neurotoxicity, CB_1_ receptor mRNA was elevated in the globus pallidus and subthalamic nucleus in response to levodopa-induced dyskinesia (Rojo-Bustamante et al., 2018). CB_2_ receptor mRNA abundance was also elevated in the substantia nigra but diminished in the caudate putamen in human and mouse tissues (Gómez-Gálvez et al., 2016; Navarrete et al., 2018). Immunofluorescence labeling of human PD brains supported that the elevation in CB_2_ receptors was primarily in activated microglia within the substantia nigra pars compacta (Gómez-Gálvez et al., 2016). In the MPTP-induced neurotoxicity model of PD, Price et al. (2009) identified elevated levels of CB_2_ receptor protein in the ventral midbrain via western blot and immunofluorescence. This labeling colocalized with CD11b/CD18^+^ cells which indicated that the CB_2_ receptors were indeed expressed in activated microglia. In the reserpine-induced animal model of PD, a substantial increase in 2-AG and AEA was observed in the globus pallidus (Di Marzo et al., 2000). A similar increase in 2-AG was also found in the mouse ventral midbrain using an MPTP-treated mouse model (Mounsey et al., 2015). Elevated AEA was also found in the basal ganglia of rats lesioned with 6-hydroxy dopamine (6-OHDA) (Maccarrone et al., 2003). This was accompanied by reduced FAAH activity in the striatum. Thus, endocannabinoid production appears to be elevated in PD, perhaps as a compensatory mechanism to dampen the associated neuroinflammation.

Activation of microglial CB_2_ receptors has been shown to be neuroprotective and improve motor symptoms in several animal models of PD (Price et al., 2009; Chung et al., 2016; Cassano et al., 2017). A naturally occurring CB_2_ receptor agonist, β-caryophyllene (BCP), was neuroprotective and dampened the pro-inflammatory response of microglia in rats in a rotenone-induced model of PD (Javed et al., 2016; Ojha et al., 2016). Administration of WIN55,212-2 reduced neuronal death and improved motor symptoms in mice subjected to MPTP-dependent neurotoxicity (Price et al., 2009). Treatment with WIN55,212-2 also reduced the number of M1 microglia in the ventral midbrain. An equal effect was observed within the same study upon administration of a CB_2_ receptor-selective agonist, JWH-015. Interestingly, the effects of WIN55,212-2 on microglial activation were completely blocked by the CB_2_ receptor-selective inverse agonist, JTE-907. These results were unchanged in CB_1_ receptor knockout mice, but the MPTP-dependent neurotoxicity was exacerbated in CB_2_ receptor knockout mice. Thus, the effects of WIN55,212-2 were likely mediated solely by CB_2_ receptors despite the nonselective nature of the ligand. Taken together, CB_2_ receptors, specifically on microglia, may represent a therapeutic target to reduce neuroinflammation and protect neurons through the development of PD.

### Huntington’s Disease

HD is an inherited disorder that is characterized by the progressive loss of dopaminergic neurons in the indirect pathway of the striatum which causes locomotor and cognitive impairments (Cristino et al., 2020). An increased abundance of activated microglia has been measured in the cortex and striatum in human HD brains compared to aged humans free of neurological disorder (Sapp et al., 2001). There was also a strong positive correlation between disease stage and the accumulation of primed proinflammatory microglia as measured by the abundance of MHC class II antigens (Sapp et al., 2001). Positron emission tomography has been used to measure a marked increase in the binding of radiolabeled PK-11195 in cortical brain regions and in the striatum of patients with HD (Pavese et al., 2006; Yen F. Tai et al., 2007a). As PK-11195 is known to bind primarily to glial cells in the injured CNS, this would indicate that there was a substantial increase in the abundance of activated microglia in the human HD brain tissue (Cagnin et al., 2002). Pavese et al. (2006) also found that the degree of microglial activation correlated with disease stage which implicated a direct role of microglia in the progression of the disease. This group has used similar methodologies to determine that there was an elevated number of microglia in the striatum and cortex of pre-symptomatic carriers of the mutant *HTT* gene with abnormally expanded CAG repeats (Yen F. Tai et al., 2007b). The elevation in activated microglia was also correlated with decreased binding of ^11^C-raclopride, indicative of striatal neuron loss. These data indicate that the microglial response and neuronal dysfunction occur in tandem, several years prior to the predicted age of disease onset of HD based on the number of CAG repeats.

HD progression has been characterized by a loss of neuronal CB_1_ receptors in several transgenic mouse models as well as in post-mortem human HD brains (Glass et al., 1993; Denovan-Wright and Robertson, 2000; Lastres-Becker et al., 2002; Dowie et al., 2009; Blázquez et al., 2011). Conversely, an upregulation of CB_2_ receptors has been observed in the striatum of R6/1 and R6/2 transgenic mice as well as human HD brains (Palazuelos et al., 2009). The immunolabeling revealed colocalization with ionized calcium-binding adapter molecule 1 (Iba1) but not glial fibrillary acidic protein (GFAP) which indicated that the receptors were specifically upregulated in microglia. Male Sprague Dawley rats that received an intrastriatal injection of malonate exhibited a 4-fold increase in CB_2_ receptor mRNA within the striatum (Sagredo et al., 2009). Many of the CB_2_ receptors were expressed in activated M1 microglia, although astrocytes were also identified as CB_2_ receptor positive. However, Dowie et al. (2014) reported that upregulation of CB_2_ receptor protein was localized to the vasculature and not microglia or astrocytes in human HD brain tissue. When R6/2 mice were crossed with CB_2_ receptor knockout mice, the offspring exhibited aggravated motor symptoms which indicates that constitutive CB_2_ receptor activity was beneficial to disease progression in this model (Palazuelos et al., 2009). The striata from these R6/2 mice had higher proportions of M1 microglia with elevated IL-1β, IL-6, TNFα, and iNOS. Given these data, elevated microglial CB_2_ receptors may not have been simply induced by the pro-inflammatory state. Thus, CB_2_ receptors may regulate microglial activation and play a protective role in the context of HD.

Based on current data, it appears that CB_1_ and CB_2_ receptors play important roles in HD to control excitotoxicity and neuroinflammation, respectively. Thus, the use of therapeutics to preserve CB_1_ receptors and activate CB_2_ receptors may be a useful strategy to treat symptoms of HD. One method to preserve neuronal CB_1_ receptors appears was through stimulation of the receptors. Laprairie et al. (2013) determined that the selective CB_1_ receptor agonist, arachidonyl-2′-chloroethylamide (ACEA), upregulated neuronal expression of CB_1_ receptor mRNA and protein in the ST*Hdh* ^Q7/Q7^ and ST*Hdh* ^Q111/Q111^ cell models of HD. These effects were mediated by NF-κB and Akt downstream of CB_1_ receptor activation. Sagredo et al. (2009) found that direct stimulation of CB_1_ receptors using ACEA did not improve the survival of striatal projection neurons following an acute neurotoxic malonate lesion in Sprague Dawley rats. There was also no benefit of Δ^9^-THC or HU-210 (synthetic nonselective agonist) to preserve CB_1_ receptors in the R6/1 mouse model of HD (Dowie et al., 2010). CB_1_ receptors are also limited as a therapeutic target due to the psychoactivity associated with global receptor activation (Ashton and Glass, 2007). A method to circumvent these limitations may be to use positive allosteric modulators to enhance CB_1_ receptor activation by endogenous cannabinoids. Positive allosteric modulators of CB_1_ receptors have shown to improve cell viability in a cell model of HD as well as improve motor coordination and delay symptom onset in R6/2 mice (Laprairie et al., 2019). Furthermore, inhibition of FAAH using URB597 preserved CB_1_ receptors in the striatum of R6/1 mice (Dowie et al., 2010). The CB_2_ receptor-selective agonist HU-308 has been neuroprotective and reduced the TNFα concentration in rats that received intrastriatal malonate injections (Sagredo et al., 2009). Otherwise, there is limited data to support the use of CB_2_ receptor-selective agonists specifically in HD. However, therapeutics that target microglial CB_2_ receptors to dampen the neuroinflammatory response have been generally promising for brain diseases with an inflammatory component (Navarro et al., 2016).

### Aging

Even in the absence of disease, aging brains exhibit an elevation in the proportions of activated M1 microglia that secrete pro-inflammatory cytokines such as TNFα, IL-1β, and IL-6 in the central nervous system; proportions of M2 microglia are also reduced which leads to a deficiency in anti-inflammatory cytokines such as IL-10 (Ye and Johnson, 1999; Ye and Johnson, 2001; Lukiw, 2004; Streit et al., 2004; Zahn et al., 2007). In brains of aged mice (∼20 months old), 25% of microglia have been reported to be MHC class II-positive compared to only 2% of microglia in healthy adult mice (∼4 months old) (Henry et al., 2009). Thus, a much larger proportion of microglia in aged mice were primed for pro-inflammatory activity compared to younger mice under otherwise healthy conditions (Norden and Godbout, 2013). A number of studies have demonstrated that aged mice are also more sensitive to inflammatory stimuli compared to adult mice (Wynne et al., 2010; Njie et al., 2012). Sierra et al. (2007) found that aged mice had an enhanced response to LPS injection and had higher expression of TNFα, IL-1β, IL-6, and IL-12 mRNA in microglia compared to adult mice. Henry et al. (2009) also demonstrated that microglia isolated from aged mice exhibited elevated mRNA abundance for proinflammatory cytokine production. There were functional consequences to the elevation of these cytokines as the increased inflammatory properties of aged brains has been associated with psychomotor and cognitive impairment in mice (Weaver et al., 2002; Richwine et al., 2005; Hayashi et al., 2008; Villeda et al., 2011). The exaggerated immune response in elderly populations has also been associated with increased susceptibility to behavioural complications following peripheral immune challenge, including depression and cognitive impairment (Godbout and Johnson, 2006; Godbout and Johnson, 2009; Corona et al., 2012).

The balance of microglial phenotypes has been found to change with age toward an increase in pro-inflammatory phenotypes, with the emergence of some transcriptional phenotypes not observed in younger mice (Hammond et al., 2019). Microglia from aged mice (24-month old) exhibited markers of pro-inflammation, including upregulation of markers of cytokine release and phagocytosis (Raj et al., 2017). Microglia from aged animals also differed substantially from those of young animals in terms of metabolism, potentially due to differences in rapamycin-insensitive companion of mTOR (RICTOR) which was a common upstream regulator of many of the dysregulated metabolic processes (Flowers et al., 2017). Interestingly, cultured BV-2 microglia in the absence of RICTOR exhibited the same phenotype as primary microglia from aged animals. This may indicate that microglial metabolic dysregulation with age can cause multiple phenotypes to converge.

As aged brains exhibit a higher proportion of pro-inflammatory microglia compared to young brains, it would be expected for aged brains to contain elevated levels of CB_2_ receptors as well. However, Hodges et al. (2020) reported no statistically significant differences in CB_2_ receptor mRNA from the cortex or hypothalamus between young and aged mice. In contrast, Pascual et al. (2014) reported decreased CB_2_ receptor abundance in aged rats. Aged rats (24-month old) exhibited a 50% reduction in CB_1_ receptor mRNA in the basal ganglia compared to young rats (3-month old) as measured by autoradiography and *in situ* hybridization (Mailleux and Vanderhaeghen, 1992; Romero et al., 1998). However, this early work did not determine whether the loss of CB_1_ receptor mRNA occurred only in neurons or in microglia as well.

Stimulation of CB_2_ receptors appeared to contribute to the control of neurogenesis in an age-dependent manner (Goncalves et al., 2008). The authors found that a DAGL inhibitor, RHC-80267, inhibited the proliferation of Cor1 neural stem cell line which highlights the importance of 2-AG signaling in the proliferation of cultured neuronal stem cells. Goncalves et al. (2008) also demonstrated that selective antagonists for both CB_1_ receptors (AM251) and CB_2_ receptors (AM630) inhibited proliferation in the same cell line which suggested that the role of 2-AG in cell proliferation could be mediated by the actions of both CB_1_ and CB_2_ receptors. These results were consistent when the experiments were repeated in 6-week, 6-month, and 20-month-old female mice. Stimulation of CB_2_ receptors *via* JWH-133 also increased the number of neurons in the subventricular zone, and the effects were most pronounced in the aged mice (Goncalves et al., 2008). Thus, the benefits of CB_2_ receptor activation could translate to an aging population.

## Potential Influence of Sex and Exercise on Endocannabinoid Function

There are sex differences in the endocannabinoid system which impacts the responses to cannabinoids. In mice, females have reported higher quantities of both CB_1_ and CB_2_ receptor mRNA relative to males (Xing et al., 2014). This may partially explain the growing body of evidence that has demonstrated a greater effect of cannabinoids in females for the treatment of pain (Craft et al., 2013; Blanton et al., 2021). Microglia in the spinal cord sensitized male mice to neuropathic and inflammatory pain *via* TLR4, but the effects were not observed in female mice (Sorge et al., 2011). It was later determined that pain hypersensitivity in female mice was mediated by adaptive immune cells and not microglia (Sorge et al., 2015). Female mice also had greater quantities of microglia in the periaqueductal gray region of the brain which is involved in descending pain modulation (Doyle et al., 2017). This difference in number of microglia was proposed to explain the sex differences in the effectiveness of morphine for pain relief. As morphine interacts with microglial TLR4 to initiate a pro-inflammatory response, this may stimulate neuroinflammation which would counteract the analgesic effects (Hutchinson et al., 2010). Therefore, there are apparent inherent sex differences in microglia with respect to pain processing. To our knowledge, the connection between sex differences in cannabinoid receptor quantities and microglial distribution has not been investigated with respect to neuroinflammation. However, this information will be critical to tailor CB_2_ receptor-targeted therapies for the treatment of neuroinflammation.

The endocannabinoid system, and especially CB_1_ receptors in peripheral tissues, become dysregulated with lifestyle related diseases such as obesity (Matias et al., 2008). High-fat diets associated with obesity lead to higher quantities of circulating endocannabinoids and increased CB_1_ receptor activation which drove increased food intake and reduced insulin sensitivity and energy metabolism in skeletal muscle (Pagotto et al., 2006). Selective CB_1_ receptor antagonists have been in development as anti-obesity agents, however, these drugs include several negative side effects which has precluded their clinical use (Quarta and Cota, 2020). Interestingly, lifestyle interventions such as consistent exercise appear to counteract the overexpression of CB_1_ receptors in peripheral tissues (Heyman et al., 2012). Consistent exercise was also associated with improvements in cognition for AD patients, although a link to endocannabinoid-mediated mechanism has not been established to our knowledge (Meng et al., 2020).

## Mechanisms of CB_2_ Receptor-Mediated Effects in Microglia

Activation of cannabinoid receptors has consistently been observed to dampen the shift of microglia to an M1 phenotype following treatment with a pro-inflammatory stimulus such as LPS or IFNγ by inhibiting the release of soluble factors including NO, TNFα, and IL-6 (Tanaka et al., 2020). Activation of CB_2_ receptors also appears to regulate the shift from an unreactive phenotype to an anti-inflammatory M2 phenotype. It is possible that cannabinoids also facilitate a shift from an M1 phenotype directly to an M2 phenotype. Although there is a clear relationship based on the profiles of cytokines released from the microglia, the specific mechanisms and signaling pathways involved have not been thoroughly examined. Recent evidence has implicated the MAPK pathways as potential targets to explain the relationship between cannabinoid signaling and inflammation.

MAPKs are intracellular signaling proteins that are responsible for many downstream functions and can be subdivided into c-Jun N-terminal Kinase (JNK), extracellular signal-regulated kinase (ERK), and p38 proteins. Each of the MAPK signaling pathways has also been associated with both the pro-inflammatory and anti-inflammatory properties of microglia (Kim et al., 2004; Waetzig et al., 2005; Bachstetter et al., 2011; Li et al., 2019; Chen et al., 2021). As both CB_1_ and CB_2_ receptors typically couple to Gα_i/o_ and Gβγ proteins, activation of these receptors typically initiates phosphorylation of downstream MAPK pathways (Bouaboula et al., 1996; Howlett et al., 2002; Komorowska-Müller and Schmöle, 2020). Correa et al. (2011) found that treatment with AEA dampened the release of pro-inflammatory cytokines IL-12 and IL-23 in a mouse model of multiple sclerosis via the JNK and ERK1/2 pathways, but the effect was only partially mediated by CB_2_ receptors. The inhibitory effects of AEA on the release of TNFα, IL-6, and IL-1β were fully blocked by a PKC inhibitor, chelerythrine, which indicated that both CB_1_ and CB_2_ receptors may have contributed via MAPK signaling (Ma et al., 2015). Recent transcriptomic data using CB_2_ receptor knockout microglia demonstrated impaired MAPK signaling which corroborated the involvement of CB_2_ receptors in these pathways (Reusch et al., 2021). Activation of CB_2_ receptors has also been shown to reduce translocation of NF-κB p65 to the nucleus, perhaps as a downstream consequence of MAPK signaling (Correa et al., 2010; Javed et al., 2016).

Pro-inflammatory stimuli such as LPS and IFNγ have been reported to initiate MAPK signaling in microglia (Frazier et al., 2012; Meng et al., 2014). CB_2_ receptor activation diminished the downstream translation of pro-inflammatory cytokine genes in cultured microglia challenged with Aβ_1-42_ (Ehrhart et al., 2005). Thus, it seems that there is negative cross-talk among CB_2_ receptor signaling and LPS- or IFNγ-dependent MAPK signaling. Although ERK phosphorylation was induced independently by LPS and CB_2_ receptor activation in cultured BV-2 microglia, co-treatment with LPS and AEA or WIN-55,212,-2 induced a much smaller effect than either stimulus alone (Eljaschewitsch et al., 2006). This appears to be caused by the induction of MAPK phosphatase (MKP)-1/2 which dephosphorylated ERK1/2. As the induction of MKP-1 occurred much faster in the presence of LPS and AEA compared to either compound alone, LPS-mediated ERK phosphorylation was blunted. The effect of AEA was partially blocked by AM251 but fully blocked by AM630 (Eljaschewitsch et al., 2006). This indicated that both CB_1_ and CB_2_ receptors may have contributed to the inhibition of microglial pro-inflammatory phenotypes. Subsequent work has corroborated that CB_2_ receptor activation induced MKP-1 and MKP-3 which inhibited ERK phosphorylation upon LPS stimulation in primary rat microglia (Romero-Sandoval et al., 2009). Thus, it seems likely that CB_2_ receptor activation attenuates LPS-induced ERK phosphorylation and downstream transcription of pro-inflammatory genes through the induction of MKP proteins. Further investigation of endocannabinoid-mediated upregulation of MKP proteins may provide important clues into how CB_2_ receptor agonism can inhibit the activation of pro-inflammatory microglia.

Acquisition of immunomodulatory M2-like properties in microglia has been observed following CB_2_ receptor-dependent MAPK signaling. Cultured BV-2 microglia treated with LPS and IFNγ demonstrated elevated release of the anti-inflammatory cytokine IL-10 release, this was enhanced by co-incubation with AEA in a dose-dependent manner (Correa et al., 2010). A similar effect was observed when the microglia were co-incubated with the CB_2_ receptor-selective agonist, JWH-133. These effects were blocked by the CB_2_ receptor-selective antagonist, SR144528, but not influenced by the CB_1_ receptor-selective antagonist, SR141716A. This indicated that the enhanced effect on IL-10 release was mediated by the activation of CB_2_ but not CB_1_ receptors. Furthermore, the effects were blocked by the MEK1/2 inhibitor, PD98059, as well as the JNK inhibitor, SP600125. However, the PI3K/Akt inhibitor, Ly294002, had no influence over the effects (Correa et al., 2010). Thus, it appears that CB_2_ receptor activation can promote the downstream release of anti-inflammatory factors such as IL-10 via the ERK and JNK MAPK pathways. This could allow microglia in a pro-inflammatory phenotype to also acquire M2-like properties following CB_2_ receptor activation.

Non-canonical cAMP-mediated signaling pathways may also contribute to the anti-inflammatory properties of cannabinoids in microglia. CB_2_ receptors generally couple to Gα_i_ proteins and do not mediate increased cAMP (Glass and Northup, 1999; Ibsen et al., 2017). However, there is recent evidence to suggest that CB_2_ receptors could couple to Gα_s_ proteins in primary human peripheral blood mononuclear cells to elevate cAMP and activate PKA (Saroz et al., 2019). In cultured primary rat microglia treated with thrombin, co-treatment with JWH-133 caused an increase in cAMP accumulation in a dose-dependent manner (Tao et al., 2016). JWH-133 treatment also increased the downstream phosphorylation of PKA as a consequence of elevated cAMP (Tao et al., 2016). This elevation in phosphorylated PKA mediated a reduction in mRNA for pro-inflammatory markers such as CD68, TNFα, IL-1β, and IFNγ. Thrombin generally binds to Gα_i_ protein-coupled receptors such as protease-activated receptor (PAR)-1 and PAR-4 to inhibit cAMP accumulation (Simonds et al., 1989). As both PARs and CB_2_ receptors typically inhibit adenylate cyclase, it appears that there is an alteration in the signaling properties when both receptors are co-activated. This observation of cAMP accumulation upon co-treatment with thrombin and JWH-133 could be an early example of Gα_s_ protein-coupled CB_2_ receptors in microglia.

## Potential Influence of CB_2_ Receptor Heteromers

**FIGURE 2:**
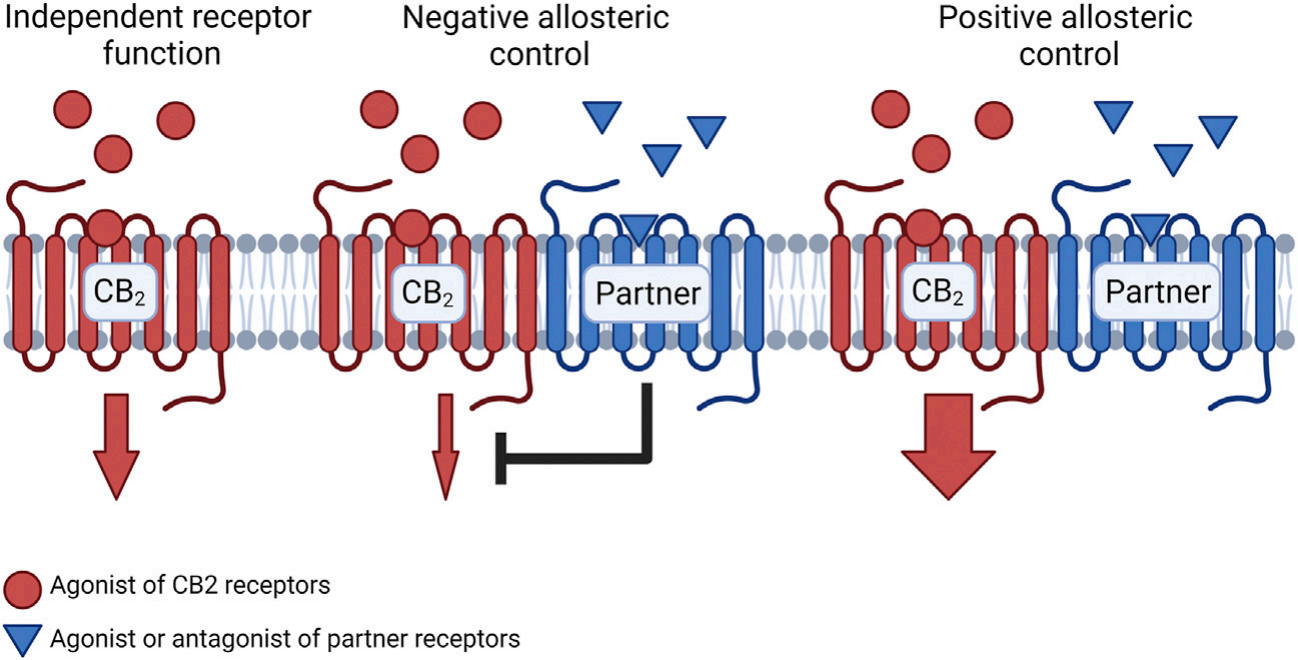
Schematic representation of signaling changes due to CB_2_ receptor heteromer formation. When presented with an agonist or antagonist for the partner receptor, the partner receptor may exert negative allosteric control over CB_2_ receptors which results in reduced signaling from the CB_2_ receptor relative to the CB_2_ receptor which does not participate in a heteromeric complex. Conversely, a partner receptor may exert positive allosteric control over the CB_2_ receptor which enhances signaling from the CB_2_ receptor mediated by the CB_2_ receptor agonist. Figure created with BioRender.

Cannabinoid receptors have been found to form oligomeric receptor complexes, known as heteromers, with several other class A GPCRs such as adenosine receptors (Carriba et al., 2007; Aso et al., 2019; Franco et al., 2019a; Köfalvi et al., 2020) and serotonin receptors (Franco et al., 2019b). Interestingly, cannabinoid receptor heteromers exhibit distinct signaling properties compared to the individual receptors alone (Callén et al., 2012; Balenga et al., 2014; Navarro et al., 2018). CB_2_-A_2A_, CB_2_-5HT_1A_, and CB_2_-CB_1_ heteromers have been observed within microglia and fundamentally alter the microglial response to cannabinoids (Navarro et al., 2018; Franco et al., 2019a; Franco et al., 2019b). Furthermore, these heteromers have been found in different quantities under conditions of neuroinflammation and in response to different cannabinoid treatments (Navarro et al., 2018; Bagher et al., 2020). Currently, there are several established heteromer-dependent mechanisms that can result in either enhanced or diminished CB_2_ receptor-mediated signaling (Figure 2). These mechanisms will be important to consider through the development of CB_2_ receptor-selective molecules for the treatment of neuroinflammation.

Cross-antagonism is a form of allosteric control within CB_2_ receptor heteromers that involves diminished signaling from the CB_2_ receptors upon antagonism of the partner receptor. This is often a bidirectional phenomenon where an antagonist for the CB_2_ receptors would also block activity of the partner receptor. For example, CB_2_ receptor-mediated Akt/PKB phosphorylation has been inhibited in the presence of the CB_1_ receptor-selective antagonist AM251 in cells that co-expressed both receptors (Callén et al., 2012). Serotonin type 1A (5HT_1A_) receptors exert similar effects as the antagonist WAY-100635 has been observed to diminish CB_2_ receptor-mediated ERK phosphorylation when co-administered with the CB_2_ receptor agonist PM224 (Franco et al., 2019b). Interestingly, adenosine type 2A (A_2A_) receptors enhanced CB_2_ receptor signaling in microglia where the receptors co-expressed (Franco et al., 2019a). Franco et al. (2019a) determined that treatment of microglia with an A_2A_ receptor antagonist (SCH58621) resulted in an enhanced effect of CB_2_ receptor-mediated cAMP inhibition compared to agonism of CB_2_ receptors alone. Ultimately, it appears that a blockade of 5HT_1A_ receptors diminishes CB_2_ receptor-mediated signaling whereas an antagonist for A_2A_ receptors may facilitate an enhanced effect of cannabinoid treatments. Given this contrast, it can be difficult to predict how CB_2_ receptor signaling could be affected in the presence of an antagonist for another GPCR that could form heteromers with CB_2_ receptors.

When CB_2_ receptor heteromers are presented with agonists for both receptors simultaneously, the effects of the ligands may produce different effects compared to either of the agonists alone. Diminished Akt phosphorylation was observed in transfected SH-SY5Y cells upon treatment with a CB_1_ receptor agonist (ACEA) and a CB_2_ receptor agonist (JWH-133) relative to treatment with either ACEA or JWH-133 alone (Callén et al., 2012). A similarly diminished effect on ERK phosphorylation has been observed upon co-treatment with JWH-133 and an A_2A_ receptor agonist (CGS-21680) (Franco et al., 2019a). In contrast, co-treatment with PM224 and a 5HT_1A_ receptor agonist (8-OH-DPAT) produced an enhanced effect on ERK phosphorylation in co-transfected cells compared to either agonist alone (Franco et al., 2019b). Thus, coactivation of CB_2_ receptors and A_2A_ receptors may diminish the effects of CB_2_ receptor agonists whereas coactivation with 5HT_1A_ receptors may lead to enhanced cannabinoid-mediated effects.

## Recent Developments and Current Challenges

It has become clear that there is an association between neurodegenerative diseases and the presence of pro-inflammatory microglia which propagate the process of neuroinflammation. However, it has been difficult to determine whether the microglia are involved in the development of these pathological conditions or simply responding to the damage. It has been proposed that microglia react to neurodegeneration to initiate neuroinflammation which exacerbates the damage, but there is emerging evidence which suggests that aberrant microglial activity could contribute to the development of such diseases. The erasure of microglia from R6/2 HD mice using a CSF1 antagonist promoted the maintenance of cognitive function and striatal neurite density and prevented the onset of some disease symptoms including loss of grip strength and striatal atrophy (Crapser et al., 2020). This would indicate that the microglia within the mouse HD brains induced damage that was ameliorated upon microglial depletion. Microglia expressing mutant huntingtin have been observed to be hyperreactive and released elevated quantities of pro-inflammatory cytokines at baseline (Crotti et al., 2014). This makes it difficult to distinguish between a potential detrimental effect of normal microglia compared to the neuroinflammation initiated by the mutant microglia. Similar benefits have been found upon depletion of microglia in AD mouse models (Spangenberg et al., 2016; Spangenberg et al., 2019). 5xFAD mice that received a CSF1 antagonist for 4 weeks maintained greater neuronal density compared to the vehicle treatment without alterations to Aβ levels (Spangenberg et al., 2016). Similar experiments were performed in younger mice prior to Aβ plaque development. These experiments revealed a lack of Aβ development in animals that received a CSF1 antagonist, however, Aβ plaques began to develop with microglial repopulation upon removal of the drug treatment (Spangenberg et al., 2019). These emerging data demonstrate that diseased microglia likely contribute to the progression of some neurodegenerative diseases, and that early targeting of these cells could be beneficial to prevent these contributions.

Several strategies have been employed to target the endocannabinoid system for the treatment of inflammation and neurodegeneration in humans. These strategies have primarily included combinations of phytocannabinoids, and synthetic CB_2_ receptor agonists. The most common method to engage the endocannabinoid system for the treatment of neuroinflammation or neurodegeneration has been with phytocannabinoids, including combinations of Δ^9^-THC and cannabidiol (CBD). Sativex™, which combines relatively equal amounts of Δ^9^-THC and CBD, has been tested in clinical trials for the treatment of HD (ClinicalTrials.gov identifier: NCT01502046). The results of the pilot cross-over trial indicated that Sativex™ was well tolerated in patients but there was no benefit to the disease progression (López-Sendón Moreno et al., 2016). Although Sativex™ has been approved for the treatment of neuropathic pain in multiple sclerosis, there is no clear clinical evidence that such phytocannabinoid-based drugs are useful specifically for the treatment of neuroinflammation in humans (ClinicalTrials.gov identifier: NCT00391079). Perhaps the most promising preclinical data has emerged from the use of synthetic selective CB_2_ receptor agonists to dampen the pro-inflammatory activity of microglia (Tanaka et al., 2020). Anabasum is a novel CB_2_ receptor agonist that is currently being trialed as an anti-inflammatory drug for use in cystic fibrosis, systemic sclerosis, dermatomyositis, and systemic lupus erythematosus (Clinicaltrial.gov identifiers: NCT02465450, NCT02465437, NCT02466243). Anabasum has recently demonstrated efficacy against a pro-inflammatory challenge in humans (Motwani et al., 2018). When ultraviolet light-killed *Escherichia coli* were injected intradermally into healthy individuals, anabasum treatment improved clearance of the pro-inflammatory stimulus and inhibited inflammation similar to prednisolone treatment. There was also enhanced biosynthesis of several pro-resolving lipid mediators (Motwani et al., 2018). This indicates that CB_2_ receptor agonists have potential to combat peripheral immune challenges in humans. Further work will be required to determine the potential effectiveness to combat inflammation in the brain.

The endocannabinoid system is emerging as a source of many therapeutically relevant targets for the treatment of inflammation and neurodegeneration. To further develop compounds to target the endocannabinoid system for clinical use, there are specific aspects of endocannabinoid function that require further attention. Great focus has been placed on the use of phytocannabinoids which engage an array of targets including CB_1_ receptors, 5HT_1A_ receptors, and TRPV1 ion channels (Howlett et al., 2002; de Almeida and Devi, 2020). Basic research in the functions of the endocannabinoid system has revealed more specific targets such as CB_2_ receptors on microglia for the treatment of neuroinflammation (Ashton and Glass, 2007). There is mounting preclinical evidence for the use of CB_2_ receptor agonists to treat chronic and acute inflammation (Komorowska-Müller and Schmöle, 2020). However, there has been very little investigation into the therapeutic window for CB_2_ receptor agonists. It is still unclear whether it could be beneficial to pre-treat with CB_2_ receptor agonists for any amount of time to delay any potential onset symptoms of neurodegenerative disease. It is also unclear if CB_2_ receptor agonists become less effective once a certain degree of neurodegeneration and microglial activation has been reached. Improving our understanding of the temporal therapeutic window of these drugs will be critical to determine their viability in an emergency or clinical setting. We also lack in our basic understanding of the functions of microglial CB_2_ receptors as well as how these functions may change throughout the human lifespan or in different microglial populations. For example, in addition to the regulation of immune activity, CB_2_ receptors have been implicated in other fundamental functions of microglia such as the regulation of phagocytosis (Ehrhart et al., 2005; Mecha et al., 2015; Guida et al., 2017). Microglia have important roles in pruning of synapses during development and disease (Stevens et al., 2007; Schafer et al., 2012). Thus, activation of CB_2_ receptors at specific times could be either beneficial or greatly detrimental to healthy brain development. Ultimately, understanding the function of CB_2_ receptors and the endocannabinoid system during these specific timeframes will be critical in the development of effective treatments that regulate microglial activity to dampen inflammation.

## Supplementary Material

Supplementary Table S1 Summary of the key changes that occur in the endocannabinoid system within several *in vivo* models or in human tissue with neurodegenerative diseases. Also noted is the impact of CB_2_ receptor activation in the animal model.
